# Trends and Patterns in Prostate Cancer Diagnostics During the Era of MRI Implementation – Real-world Evidence From a Population-based Study in the Stockholm Region, Sweden 2010–2023

**DOI:** 10.1016/j.euros.2026.03.015

**Published:** 2026-04-04

**Authors:** Jan Chandra Engel, Balram Rai, Martin Eklund, Fredrik Jäderling, Mark Clements, Tobias Nordström

**Affiliations:** a Department of Clinical Sciences at Danderyd Hospital, Karolinska Institutet, Stockholm, Sweden; b Department of Medical Epidemiology and Biostatistics, Karolinska Institutet, Stockholm, Sweden; c Department of Molecular Medicine and Surgery, Karolinska Institutet, Stockholm, Sweden; d Department of Radiology, Capio S:t Görans Hospital, Stockholm, Sweden

## Abstract

**Background and objective:**

Pre-biopsy magnetic resonance imaging (MRI) is recommended in prostate cancer diagnostics, but its impact on testing patterns and diagnostic outcomes on a population-level remains unclear.

**Methods:**

Using the Stockholm Prostate Cancer Diagnostics Register, we conducted a population-based study of men aged ≥40 yr without prior prostate cancer during 2010–2023. We described the trends in prostate-specific antigen (PSA) testing, the proportion of men with PSA ≥3 ng/ml undergoing further testing (MRI and/or biopsy), and the distribution of biopsy outcomes.

**Key findings and limitations:**

PSA testing was stable over time, with greater uptake observed in older men: in 2023, 14-yr prevalence was 76% in men aged 60–69 yr, and 84% in both those aged 70–79 and ≥80 yr. Among men with PSA ≥3 ng/ml, MRI within 1 yr increased from 3% in 2010 to 30% in 2023, while the proportion undergoing prostate biopsy declined from 23% to 16% (relative risk [RR]: 0.71; 95% confidence interval [CI]: 0.69–0.73). By 2023, 83% of biopsied men had a pre-biopsy MRI. Over the study period, biopsy outcomes improved: detection of International Society of Urological Pathology [ISUP] grade group [GG] ≥2 cancers more than doubled (RR: 2.12; 95% CI: 1.99–2.27), whereas both benign biopsies (RR: 0.66; 95% CI: 0.62–0.69) and ISUP GG 1 (RR: 0.61; 95% CI: 0.54–0.69) declined markedly.

**Conclusions and clinical implications:**

PSA testing remained prevalent, and pre-biopsy MRI use increased substantially. During this period, detection of significant cancer among biopsied men increased markedly while unnecessary biopsies and low-grade cancer findings decreased. This study provides real-world population-based data that are consistent with a trend toward increased precision in prostate cancer diagnostics in the era of MRI implementation.

## Take Home Message

Implementation of pre-biopsy magnetic resonance imaging in prostate cancer diagnostics reduced unnecessary biopsies and low-grade cancer detection, while more than doubling the detection of clinically significant cancers, demonstrating improved precision of diagnostics at a population level.

ADVANCING PRACTICE **What does this study add?**

This population-based study is the first to provide first real-world evidence of how magnetic resonance imaging (MRI) implementation has reshaped prostate cancer diagnostics on a regional scale. We show that while prostate-specific antigen testing remained stable while MRI use increased substantially. During this period we observed fewer biopsies performed, reduced overdetection of indolent cancers, and markedly increased detection of clinically significant cancers. These findings indicate that the benefits of MRI demonstrated in clinical trials translate into clinical practice, highlighting MRI as a cornerstone of modern prostate cancer diagnostics.

**Clinical Relevance**

The diagnostic pathway for prostate cancer has undergone significant transformation over the past decade. The introduction of risk calculators and MRI for both biopsy indication and targeting has enhanced diagnostic specificity, while also adding complexity compared to the traditional PSA-based systematic biopsy approach. This article explores how evidence from clinical trials has translated into real-world outcomes, particularly in terms of overdiagnosis and cancer detection. Associate Editor: Roderick C.N. van den Bergh.

**Patient Summary**

In this study, we found that prostate cancer testing was common, and testing patterns changed following the introduction of magnetic resonance imaging (MRI). The use of MRI increased significantly, whereas fewer men underwent prostate biopsy. At the same time, overdetection of insignificant cancers decreased, and detection of significant prostate cancer increased substantially. This highlights temporal changes in prostate cancer diagnostic practices and biopsy outcomes in the era of MRI use.

## 1 Introduction

Prostate cancer workup typically begins with an assessment of prostate-specific antigen (PSA) levels and is recommended for well-informed men who have an elevated risk of prostate cancer and a life expectancy of at least 15 yr [1]. Previous studies have demonstrated that PSA testing is prevalent and increases with age [2]. Traditionally, men with elevated PSA levels have been advised to undergo transrectal ultrasound (TRUS)-guided systematic prostate biopsies. However, this approach is associated with low diagnostic accuracy with high rates of benign biopsies and considerable overdiagnosis of indolent cancer [3], [4].

Magnetic resonance imaging (MRI) enables a more targeted approach and has, in clinical trials, been shown to improve detection of clinically significant cancer and reduce the number of biopsied men compared to the traditional pathway [5], [6], [7], [8]. The reporting on MRI has been systematically reviewed and standardized using Prostate Imaging Reporting and Data System (PI-RADS), further optimizing its use [9]. Accordingly, MRI has been introduced as a valuable tool and pre-biopsy MRI in men with elevated PSA, and has since 2015 been recommended by international guidelines [10]. This recommendation was incorporated into the Swedish national guidelines in 2020 [11].

Despite the growing body of evidence on benefits of using MRI in the diagnostic setting, its real-world implementation and impact on clinical outcomes have not been clearly elucidated.

This study aims to describe temporal patterns in the prostate cancer diagnostic pathway following the integration of MRI in the Stockholm region, Sweden. Using population-based data, we present data on contemporary patterns of PSA testing and examine trends in the utilization and outcomes of pre-biopsy MRI and prostate biopsies since the introduction of MRI. Furthermore, we assess how reporting based on the PI-RADS classification system has evolved over time.

## 2 Materials and Methods

### 2.1 Participants

The study population was defined as men aged ≥40 yr residing in the Stockholm region each year on December 31 from 2010 to 2023. This data was retrieved from the Swedish Population Register using the unique personal identification number. Through registry linkage with the Stockholm Prostate Cancer Diagnostics Register we collected data on PSA testing, MRI, and prostate biopsies.

The Stockholm Prostate Cancer Diagnostics Register contains all men with a PSA test registered in the Stockholm region. As of December 31, 2023, this cohort included 661 379 men. For analyses on diagnostic trends, we included all men with no prior diagnosis of prostate cancer. The National Cancer Register mandates all newly diagnosed cancers to be reported and the overall coverage is estimated around 95% [12].

### 2.2 Interventions

#### 2.2.1 PSA

There are three laboratories performing PSA analyses in the Stockholm region since 2003 (Karolinska University Laboratory [KUL], Synlab [formerly Aleris], and Unilabs). All test results are linked to the Stockholm Prostate Cancer Diagnostics Register by use of the personal identification number.

#### 2.2.2 MRI

During the study period, MRI of the prostate performed at eight clinics was defined as an MRI performed with any of a set of pre-defined keywords in the referral or report. Data was obtained through Sectra AB (Linköping, Sweden) where all images and radiology reports in the Stockholm region are stored. We extracted information on variables for our analyses from the individual written MRI reports, which were subsequently linked to the Stockholm Prostate Cancer Diagnostics Register.

The PI-RADS reporting system uses a five-tiered grading system to stratify the likelihood of significant prostate cancer on subsequent biopsy and has standardized MRI reporting [9], [13], [14]. Suspicious lesions are defined as PI-RADS 4 and 5 and are recommended MRI-targeted biopsy, whereas PI-RADS 3 lesions are considered equivocal and further workup is influenced by other clinical variables such as PSA density [15]. Lesions characterized as PI-RADS 1 and 2 are considered not suspicious of significant cancer.

#### 2.2.3 Prostate biopsies

Data on prostate biopsies were retrieved from the reports from the histopathological analyses performed at any of three existing pathology units in the Stockholm region (KUL, Synlab [formerly Aleris], and Unilabs). Biopsy outcomes were reported using Gleason score or the International Society of Urological Pathology (ISUP) grading system [16], [17]. In this study, we defined clinically significant cancer as ISUP grade group (GG) ≥2 (Gleason score ≥3+4). Data available did not discriminate between systematic and MRI-targeted biopsies, nor whether a transrectal or transperineal approach was used.

### 2.3 Statistics

We calculated the proportion of men having at least one PSA test in a calendar year from 2010–2023 by 10-yr age groups (40–49, 50–59, 60–69, 70–79, ≥80 yr). Elevated PSA, commonly defined as ≥3 ng/ml, indicates increased risk of prostate cancer and warrants subsequent MRI or systematic prostate biopsy [10]. The Swedish national guidelines largely follow European guidelines; however, they stratify PSA cut-off for further testing by age; men aged <70 yr: PSA ≥3 ng/ml; men 70–80 yr: PSA ≥5 ng/ml; men >80 yr: ≥7 ng/ml [11]. Men with metastatic disease at diagnosis or older men assessed to have very high-risk cancer are not routinely recommended MRI nor biopsy.

We assess the proportion of men with elevated PSA who underwent further diagnostics (MRI or biopsy) within 1 yr, and how these patterns have changed over time. To enable population-based estimations, this analysis includes all men with a PSA test in a given calendar year, irrespective of whether this test was a first-time or repeat measurement. Accordingly, we report the number of individuals tested per year rather than the total number of tests.

For each year, 1-yr prevalence of PSA, MRI, and prostate biopsy was calculated, and at the end of 2023 we report 14-yr prevalences. Furthermore, we evaluate the proportion of biopsied men who had performed a pre-biopsy MRI in the preceding 365 d. Additionally, we present data on the temporal trends of PI-RADS scores and biopsy outcomes by ISUP grade group.

We calculated crude relative risk (RR) and 95% confidence intervals (CIs) to present results on trends in diagnostic patterns across the study period.

The guidelines on Strengthening the Reporting of Observational Studies in Epidemiology (STROBE) [18] were used when outlining this paper (Appendix 1).

Consent was obtained from all MRI centers to access information from the written MRI reports and regional ethics committee approved the study protocol (2020-00136).

All statistical analyses were performed using SAS software, version 9.4 (SAS Institute Inc., Cary, NC, USA) and R software, version 4.5.1 (R Foundation for Statistical Computing, Vienna, Austria).

## 3 Results

From 2010 to 2023 the male population in the Stockholm region increased in all age groups, most notably in the older segments (Supplementary Table 1). Similarly, the proportion of men with a diagnosis of prostate cancer increased with age (Table 1). In 2023 a total of 615 723 men aged ≥40 yr lived in the Stockholm region and 28 547 (4.6%) had prostate cancer.

**Table 1:** Demographic presentation of diagnostic procedures in men without prior diagnosis of prostate cancer

Yrs	Population without cancer diagnosis	PSA test		PSA ≥3 ng/ml		MRI		Biopsy	
		*N*	%	*N*	%	*N*	%	*N*	%
2010	449 484	83 218	18.51	22 541	5.01	266	0.06	4506	1.00
2011	460 281	80 785	17.55	21 660	4.71	354	0.08	4523	0.98
2012	472 444	84 568	17.90	20 675	4.38	430	0.09	4700	0.99
2013	485 109	85 533	17.63	21 174	4.36	520	0.11	5137	1.06
2014	498 567	110 035	22.07	24 288	4.87	692	0.14	7166	1.44
2015	512 114	85 176	16.63	21 978	4.29	1067	0.21	4774	0.93
2016	521 175	83 832	16.09	21 299	4.09	1842	0.35	3392	0.65
2017	530 927	89 477	16.85	20 784	3.91	1894	0.36	3228	0.61
2018	540 268	92 541	17.13	21 372	3.96	2859	0.53	3434	0.64
2019	549 745	102 785	18.70	22 473	4.09	4023	0.73	2857	0.52
2020	560 345	75 428	13.46	18 043	3.22	4811	0.86	1761	0.31
2021	567 607	92 789	16.35	22 567	3.98	5461	0.96	3065	0.54
2022	576 829	93 284	16.17	22 850	3.96	7841	1.36	3821	0.66
2023	587 176	99 427	16.93	23 659	4.03	6528	1.11	3137	0.53

All numbers and proportions represent individual men rather than the number of tests in each single year. Biopsies include both systematic and targeted procedures.

PSA = prostate-specific antigen; MRI = magnetic resonance imaging.

### 3.1 PSA testing

**Fig. 1:**
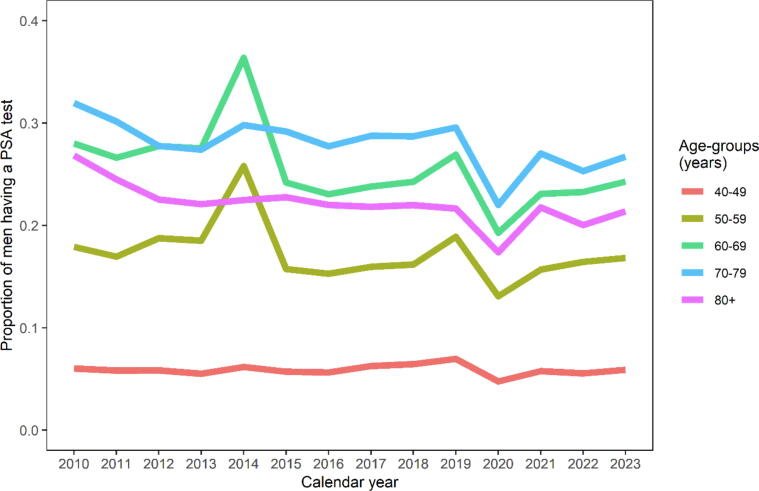
One-year prevalence of men with no prior diagnosis of prostate cancer having at least one PSA test by 10-year age-groups in the Stockholm region, Sweden.

During the length of the study period, the prevalence of PSA testing was stable, with the exceptions of when two regional screening studies were conducted in 2014 and 2019 (Fig. 1) [4], [8]. Proportions of men tested, excluding those participating in the trials, are presented in Supplementary Fig. 1. During the COVID-19 pandemic we observed markedly reduced testing rates in 2020 and 2021.

PSA testing was most common in men aged 60–69 yr and 70–79 yr with 1-yr prevalence of 24.3% and 26.7% in 2023, respectively. By the end of 2023, the 14 yr prevalence of PSA testing was 24.3% in men aged 40–49 yr, 54.6% in 50–59 yr, 75.8% in 60–69 yr, 84.1% in 70–79 yr, and 84.0% in men aged ≥80 yr.

### 3.2 MRI in men with elevated PSA

**Fig. 2:**
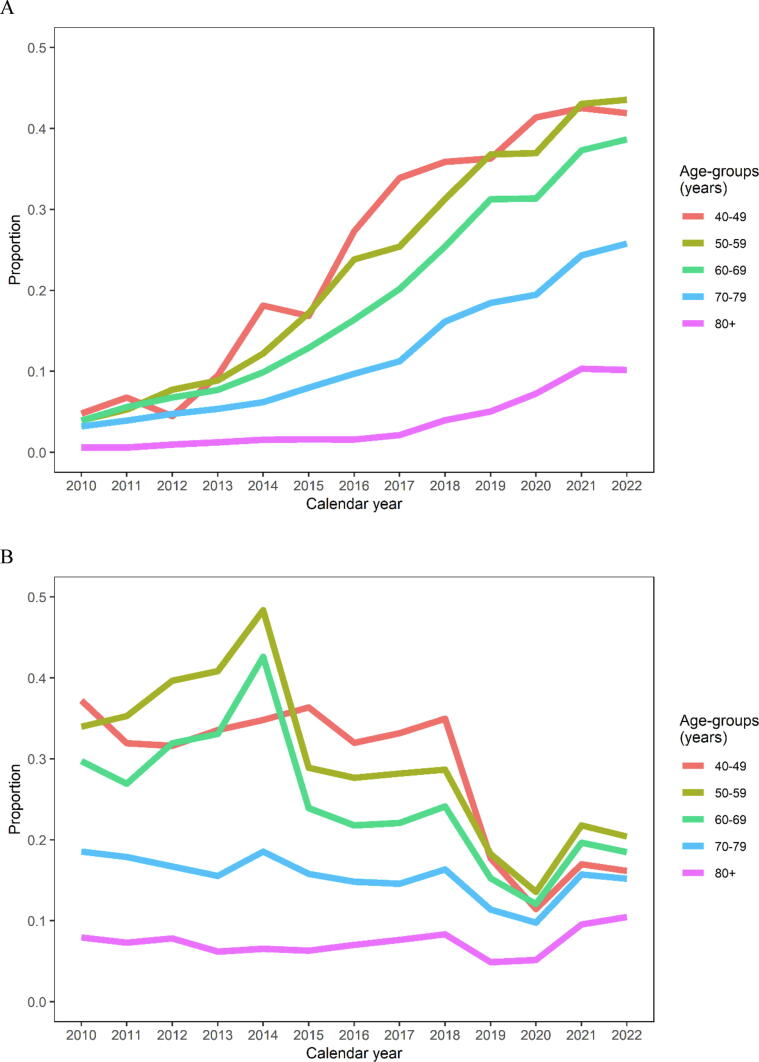
Proportion of men with PSA ≥3 ng/ml undergoing (A) MRI and/or (B) a biopsy procedure within the following 365 days. These data represent subsequent further testing after elevated PSA tests in the Stockholm region, including men with repeat PSA testing.

The use of MRI in men with PSA ≥3 ng/ml increased in all age groups; however, most notably in younger men (Fig. 2A). The uptake of MRI within a year after an elevated PSA test increased from 3.1% in 2010 to 30.1% in 2023. Although this represents a substantial increase, the overall use remains relatively low, likely reflecting the real-world setting of our study, where men often undergo repeated PSA tests; in such cases, clinicians may reasonably refrain from referring for MRI if PSA levels are elevated but stable.

**Fig. 3:**
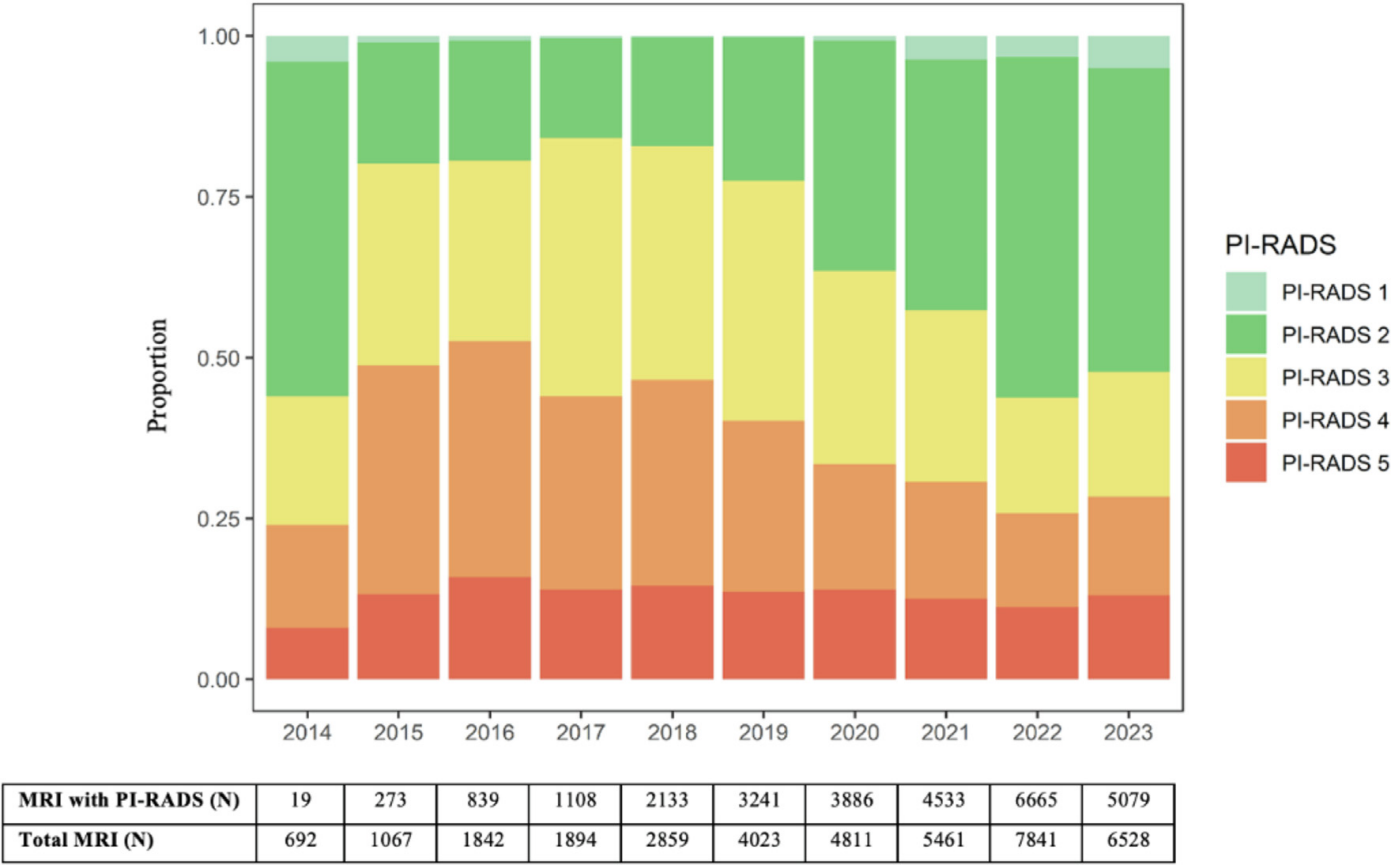
Distribution of PI-RADS scores among men ≥40 years with PSA ≥3 ng/ml undergoing MRI without a prior prostate cancer diagnosis from 2014–2023.

Reporting using the PI-RADS score system also increased markedly since its introduction in 2012, reaching 77% in 2023 from <1% in 2013 (Supplementary Table 2). The distribution of PI-RADS scores is shown in Fig. 3. We observed a shift especially with increased reporting of PI-RADS 1–2, and decreased PI-RADS 3 and PI-RADS 4, synchronous with the implementation of PI-RADS version 2.1 in 2019.

### 3.3 Prostate biopsies

The number of biopsied men without a prior prostate cancer diagnosis decreased from 4506 in 2010 to 3137 in 2023. The proportion of men with PSA ≥3 ng/ml who had biopsies performed within a year was also reduced from 22.8% to 16.2% (RR: 0.71 [95% CI 0.69–0.73]; Fig. 2B). This trend was true for all age groups but most marked in younger men.

**Fig. 4:**
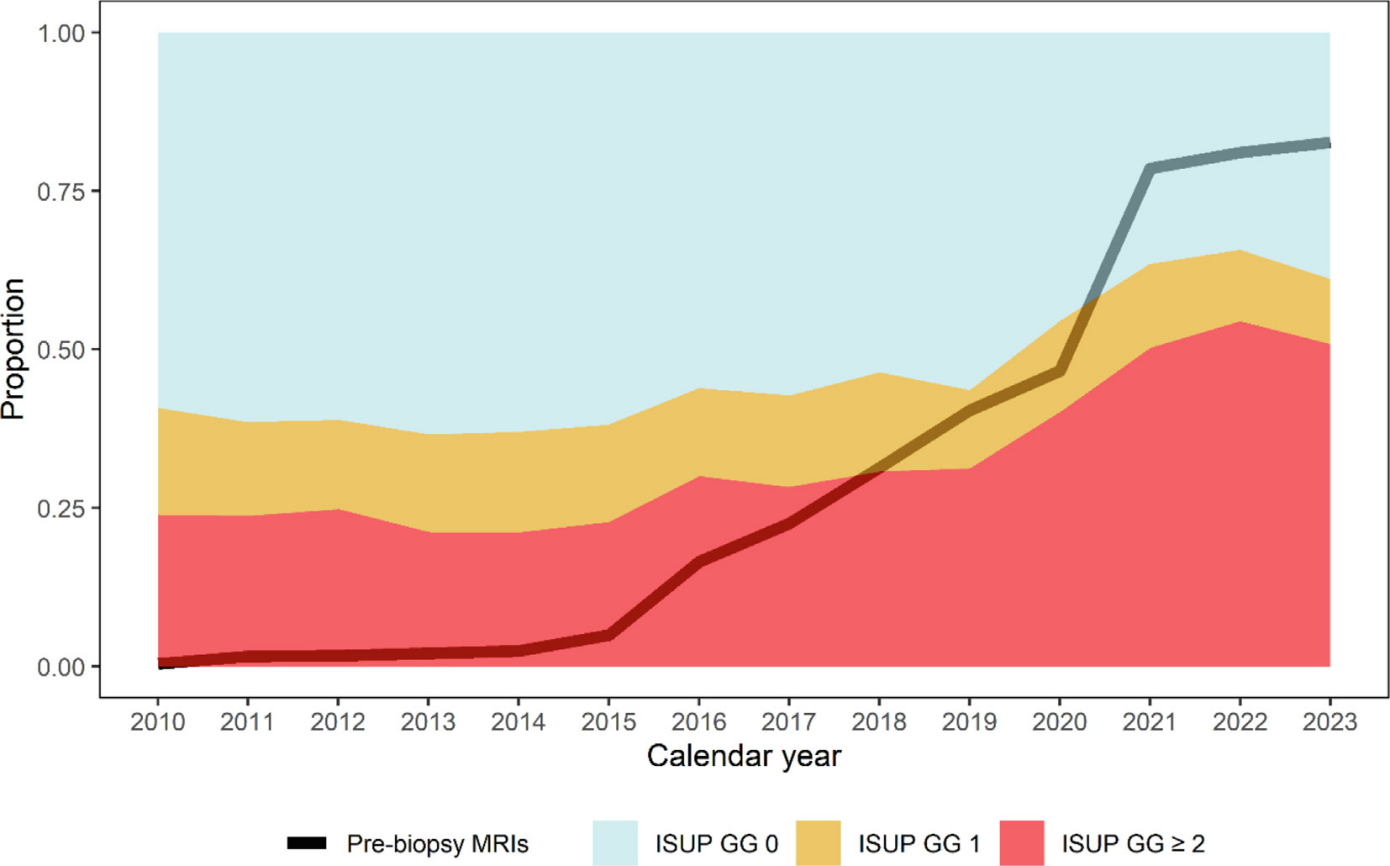
Proportion of men (≥40 years) undergoing pre-biopsy MRI and the distribution of biopsy outcomes among men undergoing a biopsy without a prior prostate cancer diagnosis, 2010–2023 in the Stockholm region, Sweden. ISUP GG: International Society of Urological Pathology grade group. ISUP GG 0 represent benign biopsy findings.

The distribution of biopsy outcomes is illustrated in Fig. 4. The proportion of benign biopsies decreased from 59% in 2010 to 39% in 2023 (RR: 0.66 [0.62–0.69]), and ISUP GG 1 from 17% to 10% (RR: 0.61 [0.54–0.69]). Detection of ISUP GG ≥2 on biopsy increased from 24% to 51% (RR: 2.12 [1.99–2.27]) during the same period.

Of all men who underwent prostate biopsies, the proportion who had had an MRI prior to the biopsy procedure increased from 0.3% in 2010 to 82.6% in 2023.

In Supplementary Fig. 2, we show results using the age-dependent PSA cut-offs for further testing as recommended by the Swedish national guidelines. When conforming to these recommendations, we observe an expected increase in the proportion of men aged ≥70 yr that underwent biopsy.

## 4 Discussion

Unnecessary biopsies and overdetection of indolent cancer remain major challenges in prostate cancer screening. MRI has been shown to improve the selection of men for biopsy and diagnostic specificity of clinically significant cancer [5], [6], [7], [8], [19], [20]. However, the population-level impact of incorporating MRI into the diagnostic chain remains unclear. This study provides unique real-world population-based data on patterns, trends, and outcomes in prostate cancer diagnostics.

Our findings show that PSA testing is common in the Stockholm region across all age groups, with more than half of all men aged >40 yr and four out of five men aged >60 yr having been tested at some point. Similar trends of increasing PSA uptake have been reported in previous studies [21], [22], [23], [24], [25]. Notably, our data indicate that PSA testing rates have remained relatively consistent over the past decade, suggesting a stable interest in the male population in early detection of prostate cancer.

Despite evidence of potential benefits, PSA screening remains controversial. Current guidelines instead emphasize individualized risk assessment, balancing the potential harms and benefits of testing and treatment [11], [26]. Our findings reveal disproportionately high PSA testing rates among older men. This pattern is inconsistent with efforts to minimize overdiagnosis and is concerning, as older men are unlikely to benefit from screening and are exposed to greater risk of unnecessary diagnosis, anxiety, and treatment-related harm [27], [28]. Restricting early detection to men who potentially could benefit from such testing is imperative to making any future screening program efficient.

Participation in organized prostate cancer testing is another challenge. Clinical trials and the Swedish organized testing program have shown acceptance rates of only 33%–50% [7], [8], [29], [30]. One possible explanation is the high level of background PSA testing demonstrated in our study. Therefore, when considering future screening initiatives it is essential to account for existing testing patterns in the target population. Our findings indicate that men’s willingness to engage in early detection is already substantial, suggesting that an introduction of any organized screening program may have limited impact on the overall number of men initiating testing. Accordingly, the benefit of an organized program may lie less in increasing uptake and more in providing structured follow-up and equitable access.

The most notable development in our data encompassing the complete diagnostic chain is the rapid increase in the use of MRI, particularly among younger men. By 2023, 83% of biopsied men had undergone an MRI prior to biopsy. Structured MRI reporting using PI-RADS, which reduces inter-reader variability and improving the quality of radiological assessment [31], was increasingly adopted after 2013. In recent years, we observed a shift in the reporting patterns with more PI-RADS 1 and 2 lesions and fewer PI-RADS 3 lesions. In 2023, more than half of all MRIs were classified as PI-RADS 1–2, which most commonly enables men to avoid prostate biopsies, illustrating how MRI was used to guide biopsy referral in routine clinical practice. This also suggests that further preselection of men prior to imaging could reduce the number of potentially unnecessary MRIs. It is also likely that PI-RADS 1–2 assessments are underestimated in our data, as radiologists have increasingly reported ‘no suspicious lesions’ instead of explicitly assigning PI-RADS 1 or 2 in the written MRI reports.

Between 2010 and 2023, both the proportion of men with PSA ≥3 ng/ml undergoing biopsy, and the number of benign biopsies were reduced by about one-third. Overdetection of ISUP GG 1 decreased by 39%, while we observed a more than twofold increase in the proportion of ISUP GG ≥2. These findings are in alignment with a multitude of previous studies supporting MRI as an effective risk stratification tool, with the potential to reduce unnecessary biopsies and overdetection of indolent cancers [5], [6], [8], [10], [19], [20], [32]. Taken together, these population-level temporal patterns are consistent with a trend toward increased precision in prostate cancer diagnostics during the era of MRI implementation, while recognizing that changes in biopsy selection and sampling contribute to the observed distributions.

Beyond improved diagnostic accuracy, these improvements also carry important implications for healthcare systems. Evidence suggests that adopting a risk-adapted strategy, leading to fewer unnecessary biopsy procedures and reduced rates of overdetection of low-risk cancer, not only optimizes patient outcomes but also contributes to more efficient resource utilization and a reduction in overall healthcare costs [33]. In this context, the population-level patterns observed in our study are consistent with a shift toward more efficient use of healthcare resources in prostate cancer diagnostics, although causal inference is not possible in this descriptive analysis.

### 4.1 Strengths and limitations

To the best of our knowledge, our study is the first population-based study describing the contemporary trends and outcomes of the complete diagnostic chain in prostate cancer, and we believe that this database can be used to monitor variations in prostate cancer diagnostics continuously.

This study also has some limitations. First, this analysis includes both first-time and repeat PSA tests. Some men may have previously undergone MRI or biopsy, reducing the likelihood of further testing after a repeated PSA, which may partly explain the relatively low proportions observed. Moreover, to avoid overdispersion of testing of men, diagnostic activity was assessed at the individual level using any subsequent testing as an indicator, rather than accounting for multiple tests per individual in a given year. While restricting the analysis to first-time tests could yield higher rates of subsequent diagnostics, it would compromise the ability to produce accurate population-based estimates. Second, due to the observational and descriptive nature of this study, causal inference is not possible. Because diagnostic practices have changed over time with increasing MRI use, biopsy outcome distributions might be subject to ascertainment bias and are not directly comparable across calendar years. In this context, it should also be acknowledged that an MRI-targeted–only biopsy strategy may contribute to grade migration. More precise sampling of small lesions can lead to increased proportion of biopsy cores with higher-grade tumors as compared with systematic biopsies. Consequently, the higher yield of ISUP GG ≥2 cancers observed in our study may partly reflect differences in sampling technique rather than true changes in disease prevalence. Finally, it cannot be excluded that a small number of men underwent PSA testing, MRI, or biopsy outside the Stockholm region. Nevertheless, we find it unlikely that this potential underreporting would have a significant effect on the actual testing rates, and even less so on the observed trends.

## 5 Conclusion

PSA testing is common and has been consistent over time also in older men where the benefit of testing remains uncertain. The use of MRI has increased substantially, and by 2023 most men had had an MRI performed prior to biopsy. Over the study period, detection of clinically significant prostate cancer increased markedly whereas rates of unnecessary biopsies and indolent cancer were reduced. Overall, these findings are consistent with a trend toward increased precision in prostate cancer diagnostics during the era of MRI implementation in routine clinical care.

  ***Author contributions***: Jan Chandra Engel had full access to all the data in the study and takes responsibility for the integrity of the data and the accuracy of the data analysis.

  *Study concept and design*: Engel, Nordström, Eklund, Jäderling, Clements, Rai.

*Acquisition of data*: Engel, Jäderling, Rai.

*Analysis and interpretation of data*: Rai, Clements Engel, Nordström.

*Drafting of the manuscript*: Engel, Nordström, Eklund, Rai, Jäderling, Clements.

*Critical revision of the manuscript for important intellectual content*: Engel, Nordström, Eklund, Rai, Jäderling, Clements.

*Statistical analysis*: Rai, Clements, Engel.

*Obtaining funding*: Nordström.

*Administrative, technical, or material support*: Nordström, Eklund.

*Supervision*: Nordström, Clements, Jäderling, Eklund.

*Other* (specify): None.

  ***Financial disclosures:*** Jan Chandra Engel certifies that all conflicts of interest, including specific financial interests and relationships and affiliations relevant to the subject matter or materials discussed in the manuscript (eg, employment/affiliation, grants or funding, consultancies, honoraria, stock ownership or options, expert testimony, royalties, or patents filed, received, or pending), are the following: None.

  ***Funding/Support and role of the sponsor*:** The name of the organization or organizations which had a role in sponsoring the data and material in the study are also listed below: Grants received from Swedish Research Council (Vetenskapliga Rådet), Prostatacancerförbundet and The Swedish cancer society (Cancerfonden).
