# Supplementary material for: Trends and Patterns in Prostate Cancer Diagnostics During the Era of MRI Implementation – Real-world Evidence From a Population-based Study in the Stockholm Region, Sweden 2010–2023
Source: Eur Urol Open Sci. 2026 Apr 4;87:48–56. doi: 10.1016/j.euros.2026.03.015 (PMC13090314; doi:10.1016/j.euros.2026.03.015)
Supplement: Supplementary Data 1 [file mmc1.pdf]

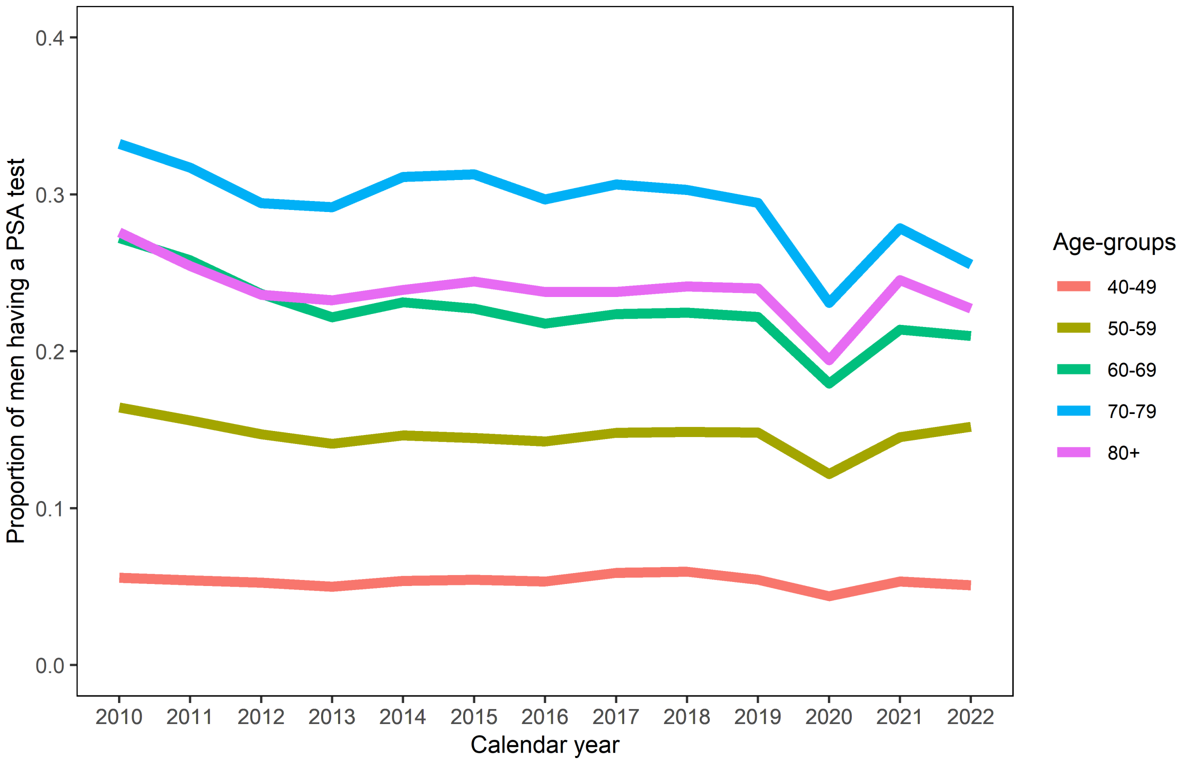

Supplementary figure 1. One-year prevalence by calendar year of men having at least one PSA test by 10-year age-groups (conditioned on men not participating in the population-based diagnostic STHLM3 and STHLM3-MRI studies)
