# Supplementary material for: Trends and Patterns in Prostate Cancer Diagnostics During the Era of MRI Implementation – Real-world Evidence From a Population-based Study in the Stockholm Region, Sweden 2010–2023
Source: Eur Urol Open Sci. 2026 Apr 4;87:48–56. doi: 10.1016/j.euros.2026.03.015 (PMC13090314; doi:10.1016/j.euros.2026.03.015)
Supplement: Supplementary Data 2 [file mmc2.pdf]

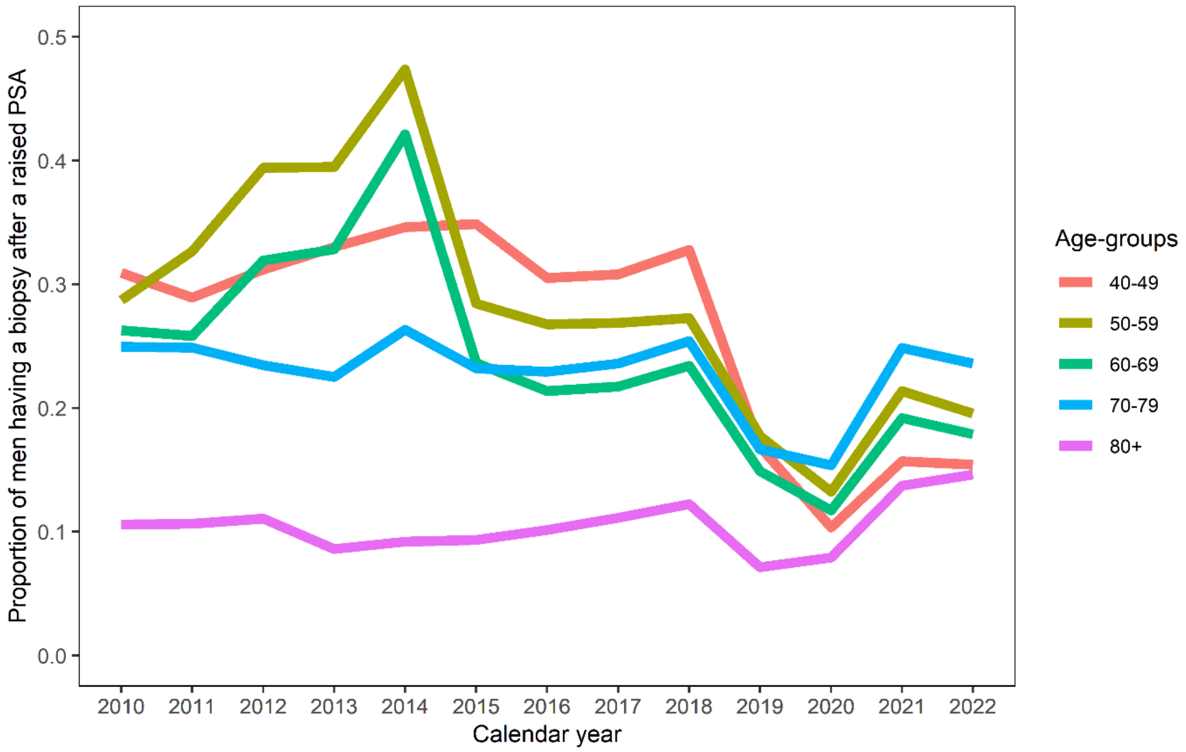

Supplementary figure 2. Proportion of men with an age-stratified elevated PSA value (men 40-69 years PSA ≥3 ng/ml, 70-79 years ≥5 ng/ml and ≥80 years ≥7 ng/ml) undergoing prostate biopsy in the following 365 days.
