# Supplementary material for: Trends and Patterns in Prostate Cancer Diagnostics During the Era of MRI Implementation – Real-world Evidence From a Population-based Study in the Stockholm Region, Sweden 2010–2023
Source: Eur Urol Open Sci. 2026 Apr 4;87:48–56. doi: 10.1016/j.euros.2026.03.015 (PMC13090314; doi:10.1016/j.euros.2026.03.015)
Supplement: Supplementary Data 3 [file mmc3.docx]

## **Age group 40-49 years**

|  | **Men without prostate cancer** | **PSA tested** | | **PSA ≥3 ng/ml** | | **MRI performed** | | **Biopsy performed** | |
| --- | --- | --- | --- | --- | --- | --- | --- | --- | --- |
| **Year** | **N** | **N** | **%** | **N** | **%** | **N** | **%** | **N** | **%** |
| 2010 | 147727  150962  154997  158428  161576  163762  164102  164668  165984  167923  170934  171937  172805  174139 | 8897 | 6.02 | 293 | 0.20 | 20 | 0.01 | 89 | 0.06 |
| 2011 |  | 8790 | 5.82 | 298 | 0.20 | 29 | 0.02 | 99 | 0.07 |
| 2012 |  | 9066 | 5.85 | 276 | 0.18 | 21 | 0.01 | 84 | 0.05 |
| 2013 |  | 8740 | 5.52 | 279 | 0.18 | 26 | 0.02 | 90 | 0.06 |
| 2014 |  | 9972 | 6.17 | 301 | 0.19 | 35 | 0.02 | 87 | 0.05 |
| 2015 |  | 9360 | 5.72 | 297 | 0.18 | 55 | 0.03 | 99 | 0.06 |
| 2016 |  | 9252 | 5.64 | 298 | 0.18 | 93 | 0.06 | 71 | 0.04 |
| 2017 |  | 10324 | 6.27 | 263 | 0.16 | 91 | 0.06 | 82 | 0.05 |
| 2018 |  | 10715 | 6.46 | 276 | 0.17 | 164 | 0.10 | 92 | 0.06 |
| 2019 |  | 11676 | 6.95 | 328 | 0.20 | 173 | 0.10 | 55 | 0.03 |
| 2020 |  | 8143 | 4.76 | 261 | 0.15 | 197 | 0.12 | 27 | 0.02 |
| 2021 |  | 9907 | 5.76 | 308 | 0.18 | 191 | 0.11 | 50 | 0.03 |
| 2022 |  | 9584 | 5.55 | 275 | 0.16 | 224 | 0.13 | 54 | 0.03 |
| 2023 |  | 10261 | 5.89 | 270 | 0.16 | 215 | 0.12 | 42 | 0.02 |

## **Age group 50-59 years**

|  | **Men without prostate cancer** | **PSA tested** | | **PSA ≥3 ng/ml** | | **MRI performed** | | **Biopsy performed** | |
| --- | --- | --- | --- | --- | --- | --- | --- | --- | --- |
| **Year** | **N** | **N** | **%** | **N** | **%** | **N** | **%** | **N** | **%** |
| 2010 | 117709  120116  123042  126542  131059  136763  140559  144425  147190  150041  152549  155032  157995  160678 | 21085 | 17.91 | 2612 | 2.22 | 43 | 0.04 | 700 | 0.59 |
| 2011 |  | 20363 | 16.95 | 2564 | 2.13 | 65 | 0.05 | 815 | 0.68 |
| 2012 |  | 23076 | 18.75 | 2515 | 2.04 | 91 | 0.07 | 848 | 0.69 |
| 2013 |  | 23417 | 18.51 | 2542 | 2.01 | 110 | 0.09 | 974 | 0.77 |
| 2014 |  | 33828 | 25.81 | 3215 | 2.45 | 146 | 0.11 | 1478 | 1.13 |
| 2015 |  | 21536 | 15.75 | 2609 | 1.91 | 251 | 0.18 | 906 | 0.66 |
| 2016 |  | 21466 | 15.27 | 2475 | 1.76 | 403 | 0.29 | 601 | 0.43 |
| 2017 |  | 23059 | 15.97 | 2365 | 1.64 | 429 | 0.30 | 573 | 0.40 |
| 2018 |  | 23807 | 16.17 | 2450 | 1.66 | 623 | 0.42 | 598 | 0.41 |
| 2019 |  | 28367 | 18.91 | 2694 | 1.80 | 864 | 0.58 | 470 | 0.31 |
| 2020 |  | 19948 | 13.08 | 2228 | 1.46 | 985 | 0.65 | 273 | 0.18 |
| 2021 |  | 24323 | 15.69 | 2822 | 1.82 | 1077 | 0.69 | 485 | 0.31 |
| 2022 |  | 25980 | 16.44 | 3036 | 1.92 | 1551 | 0.98 | 603 | 0.38 |
| 2023 |  | 27017 | 16.81 | 3094 | 1.93 | 1475 | 0.92 | 549 | 0.34 |

## **Age group 60-69 years**

|  | **Men without prostate cancer** | **PSA tested** | | **PSA ≥3 ng/ml** | | **MRI performed** | | **Biopsy performed** | |
| --- | --- | --- | --- | --- | --- | --- | --- | --- | --- |
| **Year** | **N** | **N** | **%** | **N** | **%** | **N** | **%** | **N** | **%** |
| 2010 | 105112  107207  108325  108779  108470  108248  107849  108037  108710  109470  110594  112488  114749  117619 | 29445 | 28.01 | 8588 | 8.17 | 93 | 0.09 | 2100 | 2.00 |
| 2011 |  | 28525 | 26.61 | 8278 | 7.72 | 137 | 0.13 | 2068 | 1.93 |
| 2012 |  | 30056 | 27.75 | 8043 | 7.42 | 180 | 0.17 | 2284 | 2.11 |
| 2013 |  | 29949 | 27.53 | 8286 | 7.62 | 216 | 0.20 | 2794 | 2.57 |
| 2014 |  | 39466 | 36.38 | 9697 | 8.94 | 287 | 0.26 | 3945 | 3.64 |
| 2015 |  | 26179 | 24.18 | 7411 | 6.85 | 441 | 0.41 | 2117 | 1.96 |
| 2016 |  | 24863 | 23.05 | 6789 | 6.29 | 756 | 0.70 | 1273 | 1.18 |
| 2017 |  | 25711 | 23.80 | 6421 | 5.94 | 817 | 0.76 | 1200 | 1.11 |
| 2018 |  | 26380 | 24.27 | 6613 | 6.08 | 1143 | 1.05 | 1307 | 1.20 |
| 2019 |  | 29472 | 26.92 | 6901 | 6.30 | 1658 | 1.51 | 1080 | 0.99 |
| 2020 |  | 21332 | 19.29 | 5536 | 5.01 | 2024 | 1.83 | 653 | 0.59 |
| 2021 |  | 25979 | 23.09 | 6717 | 5.97 | 2200 | 1.96 | 1096 | 0.97 |
| 2022 |  | 26701 | 23.27 | 7029 | 6.13 | 3104 | 2.71 | 1331 | 1.16 |
| 2023 |  | 28561 | 24.28 | 7275 | 6.19 | 2604 | 2.21 | 1103 | 0.94 |

## **Age group 70-79 years**

|  | **Men without prostate cancer** | **PSA tested** | | **PSA ≥3 ng/ml** | | | **MRI performed** | | | **Biopsy performed** | |  |
| --- | --- | --- | --- | --- | --- | --- | --- | --- | --- | --- | --- | --- |
| **Year** | **N** | **N** | **%** | **N** | **%** | **N** | | **%** | **N** | | **%** | |
| 2010 | 51044  53394  56727  61209  66377  71306  75888  79969  83395  85834  87905  88935  89574  89646 | 16309 | 31.95 | 6929 | 13.57 | 84 | | 0.16 | 1215 | | 2.38 | |
| 2011 |  | 16101 | 30.16 | 6667 | 12.49 | 98 | | 0.18 | 1176 | | 2.20 | |
| 2012 |  | 15755 | 27.77 | 6336 | 11.17 | 101 | | 0.18 | 1130 | | 1.99 | |
| 2013 |  | 16771 | 27.40 | 6618 | 10.81 | 136 | | 0.22 | 1004 | | 1.64 | |
| 2014 |  | 19783 | 29.80 | 7552 | 11.38 | 188 | | 0.28 | 1393 | | 2.10 | |
| 2015 |  | 20809 | 29.18 | 8008 | 11.23 | 268 | | 0.38 | 1378 | | 1.93 | |
| 2016 |  | 21039 | 27.72 | 8118 | 10.70 | 535 | | 0.70 | 1176 | | 1.55 | |
| 2017 |  | 23000 | 28.76 | 8243 | 10.31 | 501 | | 0.63 | 1063 | | 1.33 | |
| 2018 |  | 23944 | 28.71 | 8464 | 10.15 | 839 | | 1.01 | 1147 | | 1.38 | |
| 2019 |  | 25373 | 29.56 | 8936 | 10.41 | 1187 | | 1.38 | 1047 | | 1.22 | |
| 2020 |  | 19343 | 22.00 | 6926 | 7.88 | 1420 | | 1.62 | 674 | | 0.77 | |
| 2021 |  | 24043 | 27.03 | 8870 | 9.97 | 1654 | | 1.86 | 1139 | | 1.28 | |
| 2022 |  | 22665 | 25.30 | 8683 | 9.69 | 2540 | | 2.84 | 1380 | | 1.54 | |
| 2023 |  | 23951 | 26.72 | 8775 | 9.79 | 1801 | | 2.01 | 1062 | | 1.18 | |

## **Age group ≥80 years**

|  | **Men without prostate cancer** | **PSA tested** | | | **PSA ≥3 ng/ml** | | **MRI performed** | | | **Biopsy performed** | |  |
| --- | --- | --- | --- | --- | --- | --- | --- | --- | --- | --- | --- | --- |
| **Year** | **N** | **N** | **%** | **N** | | **%** | **N** | **%** | **N** | | **%** | |
| 2010 | 27892  28602  29353  30151  31085  32035  32777  33828  34989  36477  38363  39215  41706  45094 | 7482 | 26.82 | 4119 | | 14.77 | 26 | 0.09 | 402 | | 1.44 | |
| 2011 |  | 7006 | 24.49 | 3853 | | 13.47 | 25 | 0.09 | 365 | | 1.28 | |
| 2012 |  | 6615 | 22.54 | 3505 | | 11.94 | 37 | 0.13 | 354 | | 1.21 | |
| 2013 |  | 6656 | 22.08 | 3449 | | 11.44 | 32 | 0.11 | 275 | | 0.91 | |
| 2014 |  | 6986 | 22.47 | 3523 | | 11.33 | 36 | 0.12 | 263 | | 0.85 | |
| 2015 |  | 7292 | 22.76 | 3653 | | 11.40 | 52 | 0.16 | 274 | | 0.86 | |
| 2016 |  | 7212 | 22.00 | 3619 | | 11.04 | 55 | 0.17 | 271 | | 0.83 | |
| 2017 |  | 7383 | 21.83 | 3492 | | 10.32 | 56 | 0.17 | 310 | | 0.92 | |
| 2018 |  | 7695 | 21.99 | 3569 | | 10.20 | 90 | 0.26 | 290 | | 0.83 | |
| 2019 |  | 7897 | 21.65 | 3614 | | 9.91 | 141 | 0.39 | 205 | | 0.56 | |
| 2020 |  | 6662 | 17.37 | 3092 | | 8.06 | 185 | 0.48 | 134 | | 0.35 | |
| 2021 |  | 8537 | 21.77 | 3850 | | 9.82 | 339 | 0.86 | 295 | | 0.75 | |
| 2022 |  | 8354 | 20.03 | 3827 | | 9.18 | 422 | 1.01 | 453 | | 1.09 | |
| 2023 |  | 9637 | 21.37 | 4245 | | 9.41 | 433 | 0.96 | 381 | | 0.84 | |

**Supplementary Table 2.** Demographic presentation of diagnostic procedures in men without prior diagnosis of prostate cancer by 10-year age groups. These data represent individual men rather than number of tests in each single year. PSA: prostate-specific antigen. MRI: Magnetic resonance imaging.
