# Supplementary material for: Trends and Patterns in Prostate Cancer Diagnostics During the Era of MRI Implementation – Real-world Evidence From a Population-based Study in the Stockholm Region, Sweden 2010–2023
Source: Eur Urol Open Sci. 2026 Apr 4;87:48–56. doi: 10.1016/j.euros.2026.03.015 (PMC13090314; doi:10.1016/j.euros.2026.03.015)
Supplement: Supplementary Data 4 [file mmc4.docx]

| Year | Total number of MRIs performed | Proportion of MRIs with PI-RADS classification |
| --- | --- | --- |
| 2013 | 520 | <0.01 |
| 2014 | 692 | 0.03 |
| 2015 | 1067 | 0.26 |
| 2016 | 1842 | 0.46 |
| 2017 | 1894 | 0.58 |
| 2018 | 2859 | 0.75 |
| 2019 | 4023 | 0.81 |
| 2020 | 4811 | 0.81 |
| 2021 | 5461 | 0.83 |
| 2022 | 7841 | 0.85 |
| 2023 | 6528 | 0.77 |

**Supplementary Table 2.** Proportion of MRIs reported with a PI-RADS score in the Stockholm region, 2013-2023. The total number of MRIs represent the number men having an MRIs performed within one year after an elevated PSA test result in a given year.
